# Two-Year-Old Children Expect Native, but Not Foreign Speakers to Use the Same Tool for the Same Purpose

**DOI:** 10.3389/fpsyg.2021.675595

**Published:** 2021-08-17

**Authors:** Réka Petõ, Katalin Oláh, Ildikó Király

**Affiliations:** ^1^Department of Cognitive Psychology, Institute of Psychology, Eötvös Loránd University, Budapest, Hungary; ^2^MTA-ELTE Social Minds Research Group, Eötvös Loránd University, Budapest, Hungary; ^3^Cognitive Development Center, Central European University, Budapest, Hungary

**Keywords:** culture, language, cultural learning, function learning, shared knowledge

## Abstract

Previous research has already demonstrated that even very young children are sensitive to language cues and learn differently from native and foreign speaker models. A possible explanation for this phenomenon suggests that spoken language is a sign of someone's cultural background and in this sense demonstrates the person's culture specific knowledge. The aim of the present study was to investigate what children think about native and foreign speakers' behavior in a domain that is typically regulated by cultural norms (tool usage), specifically whether they expect group members to act alike or not. In a violation of expectation paradigm, two-year-old toddlers first watched a video on which a native and a foreign speaker person used different tools for achieving the same goal. In the test phase a new native speaker model appeared and selected one of the previously seen tools for the same goal as it was used before. Results indicated that toddlers were surprised if the native speaker model had chosen the tool that had previously been used by the foreign speaker. In Experiment 2, the familiarization phase was exactly the same as in Experiment 1, but during the test phase, the model spoke a foreign language. Results, in this case, showed no significant differences between looking times. These experiments suggest that two-year-olds expect native (but not foreign) speakers to use the same tool for the same goals. As tool usage is a fundamental element of cultural knowledge, we propose that this pattern of results suggest that children expect native speakers to possess shared cultural knowledge at least in the domain of artifacts.

## Introduction

Children are surrounded by an endless flow of information of which they have to select the relevant pieces that should be inevitably learned in order to become a reliable member of a given society. Recent studies provided evidence not only that social category formation helps in establishing and maintaining social interactions and in predicting obligations and expectations (Rhodes and Chalik, 2013), but also that language based social category formation plays a central role in the selection of whom to learn from. While social categories may be represented in an endless number of ways, certain distinctions carry more relevance for social learning than others. It has been suggested that linguistic group membership may be privileged in this respect, since language use provides a strong cue about the cultural background of the individual and thus can be seen as a good marker of cultural knowledgeability (Kinzler et al., 2012; Esseily et al., 2016; Soley and Spelke, 2016). Empirical evidence supports the idea that even young children view language cues as relevant during learning processes. For example, Begus and her colleagues found that 11 months infants pay more attention to a native speaker model because they expect that they would provide them with relevant pieces of information (Begus et al., 2016). It was also revealed that children selectively attend not only to the informant herself but also to the information that was presented by the model (Marno et al., 2016). In addition, infants selectively imitate and learn culture specific information (e.g., the proper way of doing things) from linguistic in-group members. For example, 14-month-old toddlers imitated an odd behavior (turned the light on with forehead) only if the model had previously spoken their native language, otherwise they rather chose a familiar and efficient behavior (turned the light on with hand) (Buttelmann et al., 2013). Relatedly, Oláh et al. (2016) and Peto et al. (2018) demonstrated that learning about an object's function can also be influenced by the model's spoken language at the age of three and four (Oláh et al., 2016; Peto et al., 2018).

Consequently, it seems that native speakers evoke epistemic trust in the domain of acquiring appropriate culture specific information. This assumption implies a related hypothesis: If children indeed learn selectively from native speakers because they are the reliable sources of culture specific knowledge contents, presumably, in addition to language, they also expect native speakers to share other pieces of culture specific knowledge. Regarding this, it seems that children and adults also expect that people who speak the same language should know the same songs (Soley and Aldan, 2020). Furthermore, Oláh et al. (2014) demonstrated that toddlers at the age of two made inferences from tools' functions to language use and expected people who used familiar objects in a novel, non-conventional way to be the sources of a foreign language utterance (Oláh et al., 2014). This data is in line with the above suggestion that children at a very early age think that spoken language is not only a cue to the borders of different social groups but to the persons' background cultural knowledge (e.g., the usage of tools) at the same time. On the other hand, the latter study focused only on the conventional and non-conventional usage of familiar objects, thus the question still remains: do children expect native speakers to share common knowledge of the specific functions of unfamiliar, novel objects too? Actually, the supposed expectation that other's “know” the function of objects that are unfamiliar for the native observers reveals that it could be a domain of shared knowledge.

Based on the previous assumption, the functions of artifacts represent an interesting domain to investigate commonly shared cultural knowledge. According to the teleo-functional stance (Casler and Kelemen, 2005, 2007), humans expect tools to serve a specific function and treat this function as an enduring and intrinsic property of the given object. Importantly, it is not only the physical properties of the object that determine what this assigned function will be. Physical properties constrain the usage of certain objects (e.g., forks are not appropriate to eat soup with), but they nonetheless may make the object appropriate to bring about different goals (e.g., a fork may be successfully used to comb our hair). Similarly, different objects may be appropriate for the very same purpose. In light of this, essentially it is the consensus in culture that unequivocally determines the currently appropriate *function*: a social group intentionally designed a tool for a specific function (e.g., eating) and all the members of that social group should use the tool (e.g., fork) for that purpose. In other words, affordance properties and cultural prescriptions together determine a tool's function. From another perspective, the properties of objects are constituted and exemplified via participation in culture specific practices (Sinha and Jensen de López, 2000). Consequently, function information is a vital element of cultural knowledge, which content should be known by all the members of that given culture, while people with different cultural background may have other knowledge alternatives. Taken this perspective, in which commonly shared cultural knowledge determines which tool should be used for a certain purpose and supposing that children expect native speakers to share other pieces of culturally shared knowledge (e.g., functions of artifacts), we predict they should also expect them to use the same tools for the same goals.

To investigate the question above we created an eye tracking study and utilized a violation of expectation paradigm. If children indeed expect native speakers to use the same tools for the same purpose, they should get surprised when their previous assumptions are contradicted. In Experiment 1, two-year-old children saw a short video in which two models (one spoke the children's native language while the other spoke a foreign one) appeared one after another and used different tools for slicing an apple. After the familiarization phase, a third person appeared with the previously seen two tools by her two sides. She held an apple in her hand, said one sentence in the children's native language and then reached for one of the two tools. The main idea was that children would expect native speakers to use the same tool for a certain purpose even if there is another object which could also serve to attain the very same goal. It is important to highlight, that in this way affordance or the goal *per se* cannot explain the model's choice between the two tools. We measured children's looking time and pattern during the test phase after the choice was made by the model. We predicted that if children have expectations about native speakers' culture dependent behavior (like the usage of tools), they would look at the videos differently. Children should not get surprised and would look shorter if the person chose the tool which had previously been used by the native speaker model (congruent condition) than when the person chose the tool that had previously been chosen by the foreign speaker (incongruent condition). This result would indicate that children do form assumptions about native speakers' behavior regarding artifacts and they expect them to use the same unfamiliar tool for the same goal even if there is another, obviously efficient tool as well. This result would mean that children think native speakers should act in the same manner. Furthermore, as tool usage is a fundamental element of cultural knowledge base, we would interpret this result as suggestive evidence that children do form the expectation that native speakers should possess the same culture specific knowledge content. This result would also indicate that two-year-old children expect native speakers to treat function as an enduring and intrinsic property of the tool that inevitably determines the usage of it.

We chose 2-years-olds to participate in our present studies because that is the earliest age at which the first sign of the teleo-functional stance can be detected (Casler and Kelemen, 2005), and even before this age, in their second year of life children understand the main function of objects conceptually (Freeman et al., 1980). Furthermore, Oláh et al. (2014) demonstrated that toddlers at that age have expectations about conventional tool use based on spoken language.

## Experiment 1

### Materials and Methods

#### Participants

Thirty monolingual Hungarian caucasian children (13 boys and 17 girls) between the age of 20 and 28 months (mean age = 23,69 months; *SD* = 1,186 months) participated in the experiment. Fifteen children (mean age = 23,8 months; *SD* = 0,33 months) took part in the congruent condition and the other 15 children (mean age = 23,55 months; *SD* = 0,28 months) in the incongruent one. Children were randomly assigned into the two conditions. Participant's parents signed an informed consent before the experiment.

#### Equipment

Tobii T60XL eye-tracker with Tobii Studio 3.2 software was used for presenting the video stimuli and collecting the data. The screen's size was 52^*^32 cm and 1900^*^1200 pixels.

#### Stimuli

**Figure 1:**
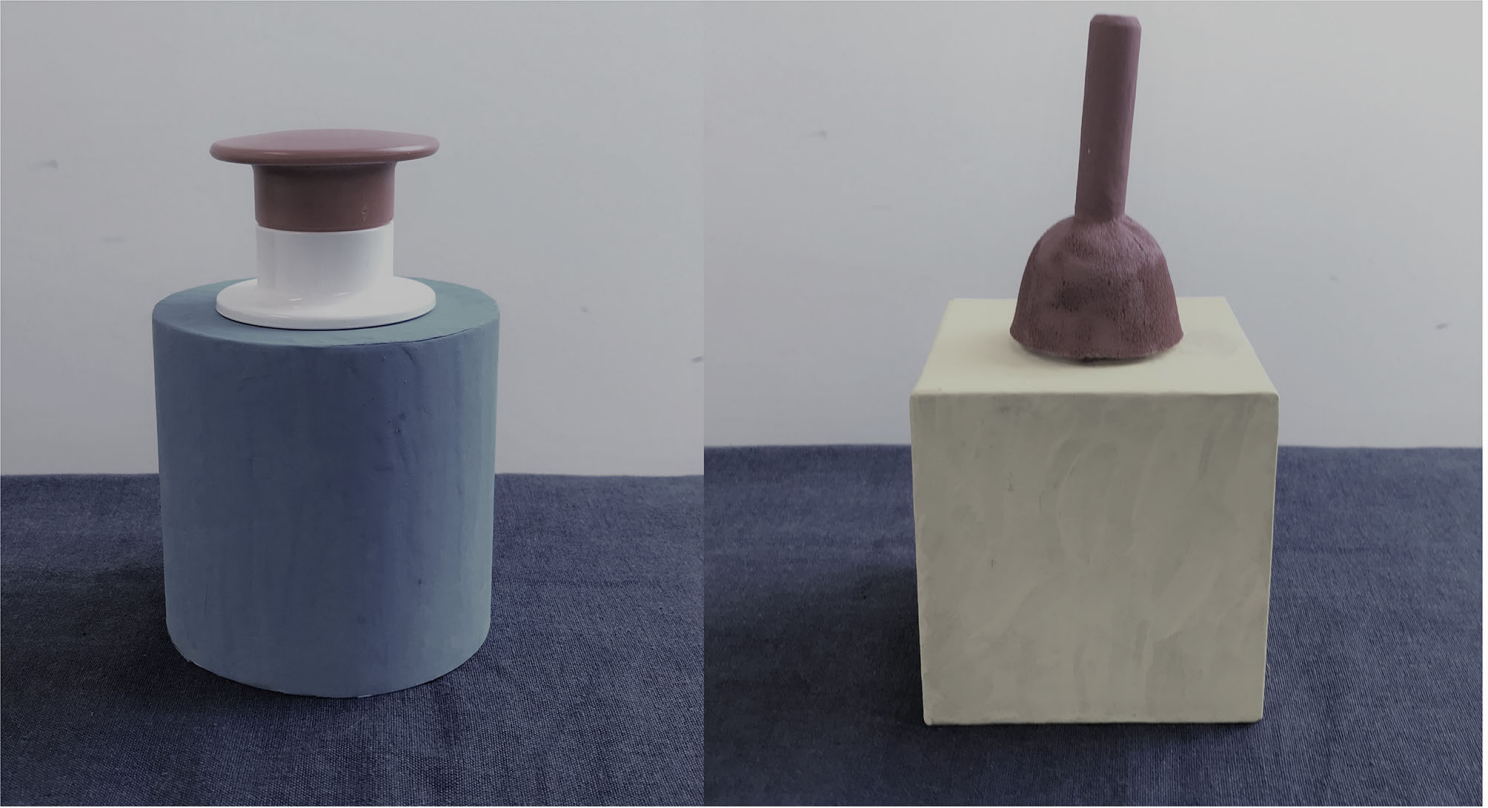
Materials. Picture depicts the objects that were used as “apple slicers” during the study both in Experiment 1 and 2.

Two novel, hand-made, functionally opaque objects (see on Figure 1) were used in the stimuli that were identically usable and successful for the same purpose: they both sliced an apple in pieces. Both of them had a button-like red piece on their top and produced the same sound after pushing it. We used novel, hand-made objects to avoid the effect of any familiarity based on children's previous knowledge. Furthermore, the application of novel objects also help to eliminate the possibility that children simply associate familiar objects' usage to familiar (native speaker) in-group members.

**Figure 2:**
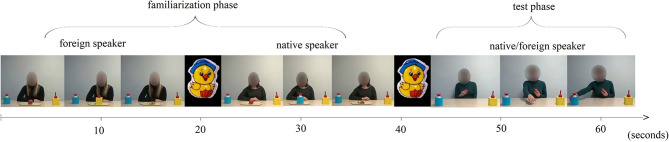
Video stimuli. Picture depicts the video that were used during the experiments. The order of appearance of the native and foreign speaker models was counterbalanced during the familiarization phase. The model during the test phase spoke either in children's native or in a foreign language.

Toddlers were presented with a 1 min 4-s-long video (see on Figure 2) that started with a 38-s-long familiarization phase. Children first saw a female who was sitting behind a table on which there was an apple in the middle and two different kinds of objects on each side of it and equal distance from the female. The model was looking at the apple for 2 s, then looked in the camera for one second and then again back to the apple and started to speak either in children's native (Hungarian) or in a foreign (Russian) language. She said one sentence: “Oh, how nice is this apple.” After that, she (without making eye contact with the child) confidently chose one of the tools and put it onto the apple. She pushed a red “button” on its top followed by a 4-s-long sound which was designed to symbolize that the tool is in operation. The female took off the tool when the sound was over and the apple appeared in slices. She looked at the apple and then started to smile and looked contentedly in the camera. Afterwards, another person appeared who repeated the very same procedure but spoke in a different language than the first model did and chose the other object to achieve the same goal. The order of appearance of the native and foreign speaker models was counterbalanced during the familiarization phase and each model chose the same object in each case. The native speaker model always picked the blue one while the foreign speaker always used the yellow one. A 1,5 s long attention-grabbing stimuli split the native and the foreign speaker model scenes.

The familiarization phase was followed by the test phase in which an unknown person appeared who was sitting behind the table, already held an apple in her hand, and the two objects were on each side of the table, equal distance from the person. The person was watching the apple, then looked up into the camera and said a sentence in children's native language: “My name is Olga, I am 25 years old.” She again looked at the apple, put it into her other hand, said “hmmm” and extended her free hand in the middle of the table, showing that she was trying to reach one of the two tools. The video was frozen for 3 s when the person's hand was right between the two objects. Afterwards the video continued and the person touched one of the objects. The person stayed in this position (her hand making contact with the object) for 10 s and children's looking time and pattern was measured within this 10-s-long time-window. A 1,5 s long attention-grabbing stimuli split the familiarization and the test phase.

Note, the female model during the test phase always spoke the children's native language and either chose the tool which had previously been used by the native speaker model (congruent condition) or the tool which had previously been used by the foreign speaker model (incongruent condition). Consequently, congruent and incongruent conditions were, respectively, defined by a choice test that either matched between the two native speakers' tool choice or did not.

#### Procedure

The experimenter welcomed the child and the parent in the laboratory. While the child explored the room and the toys, the parent signed the informed consent. After that, the experimenter escorted them to the eye-tracker room where the child sat on the parent's lap at around 60–70 cm away from the screen. The video was controlled from outside and the parent was asked to close their eyes to prevent any accidental influence on the experiment. A five point child-type calibration was used before starting each experiment. Children could watch the video stimuli only if the calibration process was successful.

#### Data Analysis

For analyzing the attentiveness during the familiarization phase, we drew an area of interest region around the entire relevant region that included the two tools and the model. We inserted a cut-off point between 0 and 38 s that included the whole familiarization phase and we measured the total visit duration indicator in both conditions.

To test whether children's looking pattern differed between the congruent and incongruent conditions we analyzed the test phase in which the model made her tool choice. We used the same AOI region as we used to measure the attentiveness during the familiarization phase. We inserted a cut-off point, set between 54 s and 1 min 4 s which covered the whole test phase after the model had made her choice. We analyzed total visit duration again to get information about children's looking time in each condition. We hypothesized that children will look longer in the incongruent condition.

In the same time-window, as a follow-up measurement we analyzed visit counts on each objects as well. For analyzing this data we drew two new AOI regions exclusively around the two objects. Visit count indicates that participants alternated their gaze between the chosen and the non-chosen object. We hypothesized that if children have a clear expectation about the model's choice, and the choice made by the model does not fulfill this expectation, not only do they look longer, but it is also likely that they would look over to the non-chosen, otherwise expected-to-be-chosen object, as an exploratory behavior. This additional measure could help us in interpreting the longer looking as a violation of expectation regarding the tool choice. Thus, children were expected to look over to the not chosen object in the incongruent condition, and not in the congruent condition.

We also attempted to analyze children's first gaze in the 3-s-long time-window when the model extended her arm but have not reached the tool yet. In this situation, our stimuli did not induce anticipatory looks (all children kept looking at the model and shifted their gaze to the objects only after the model had reached them), therefore such analyses were not performed.

#### Results

Statistical analyses were executed with the help of SPSS 17.0 Software. Children on average watched the familiarization phase for 36.38 s (*SD* = 3.7 s) on which, based on Mann-Whitney U Test, condition did not have an effect (*U* = 92; *p* = 0.395; Cohen's *d* = 0.409).

**Figure 3:**
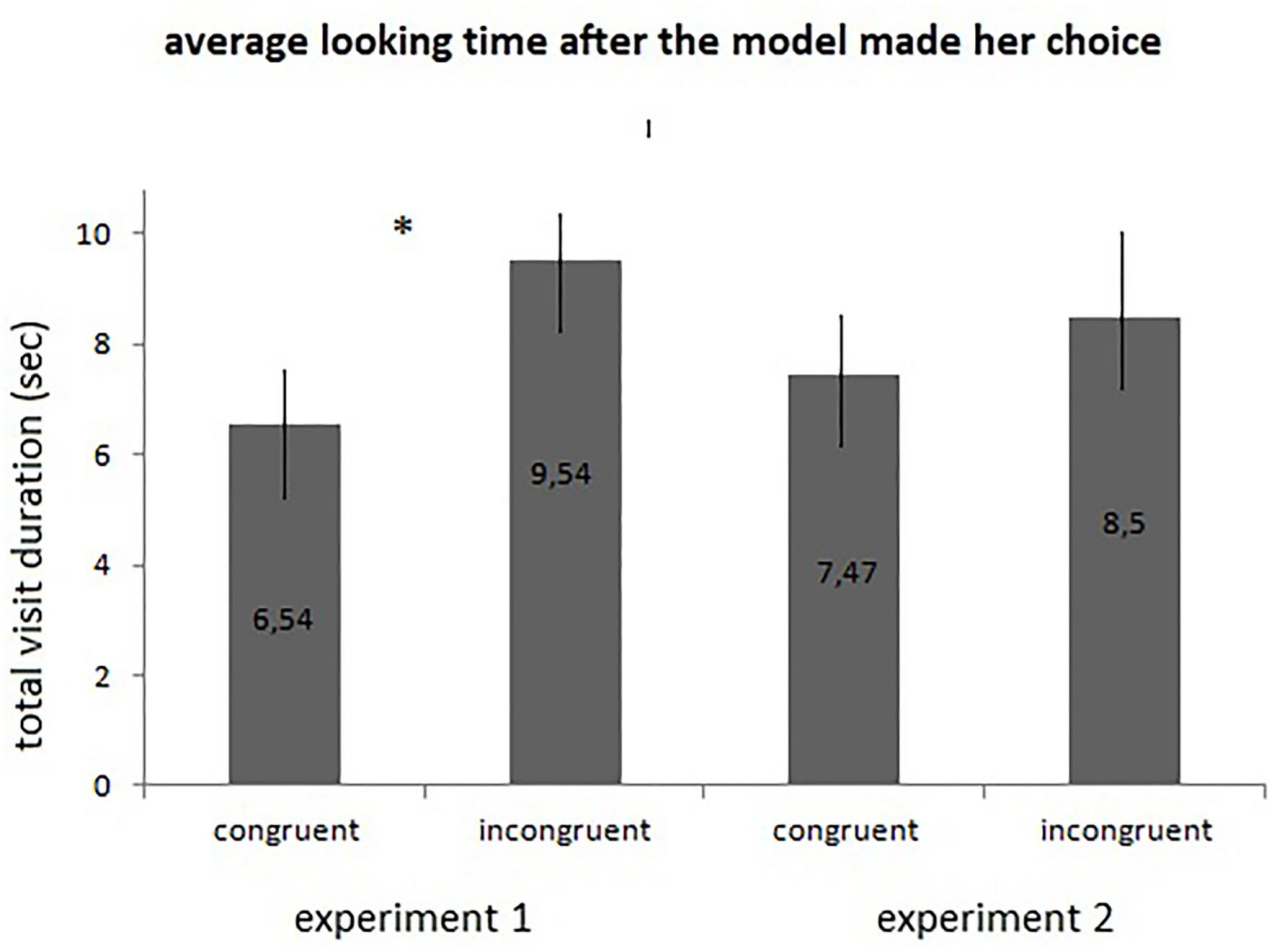
Looking times for experiment 1 and 2. This graph depicts the average looking time after the model made her choice. The asterisk indicates the significant difference which was revealed by Mann-Whitney U Test in case of Experiment 1.

**Figure 4:**
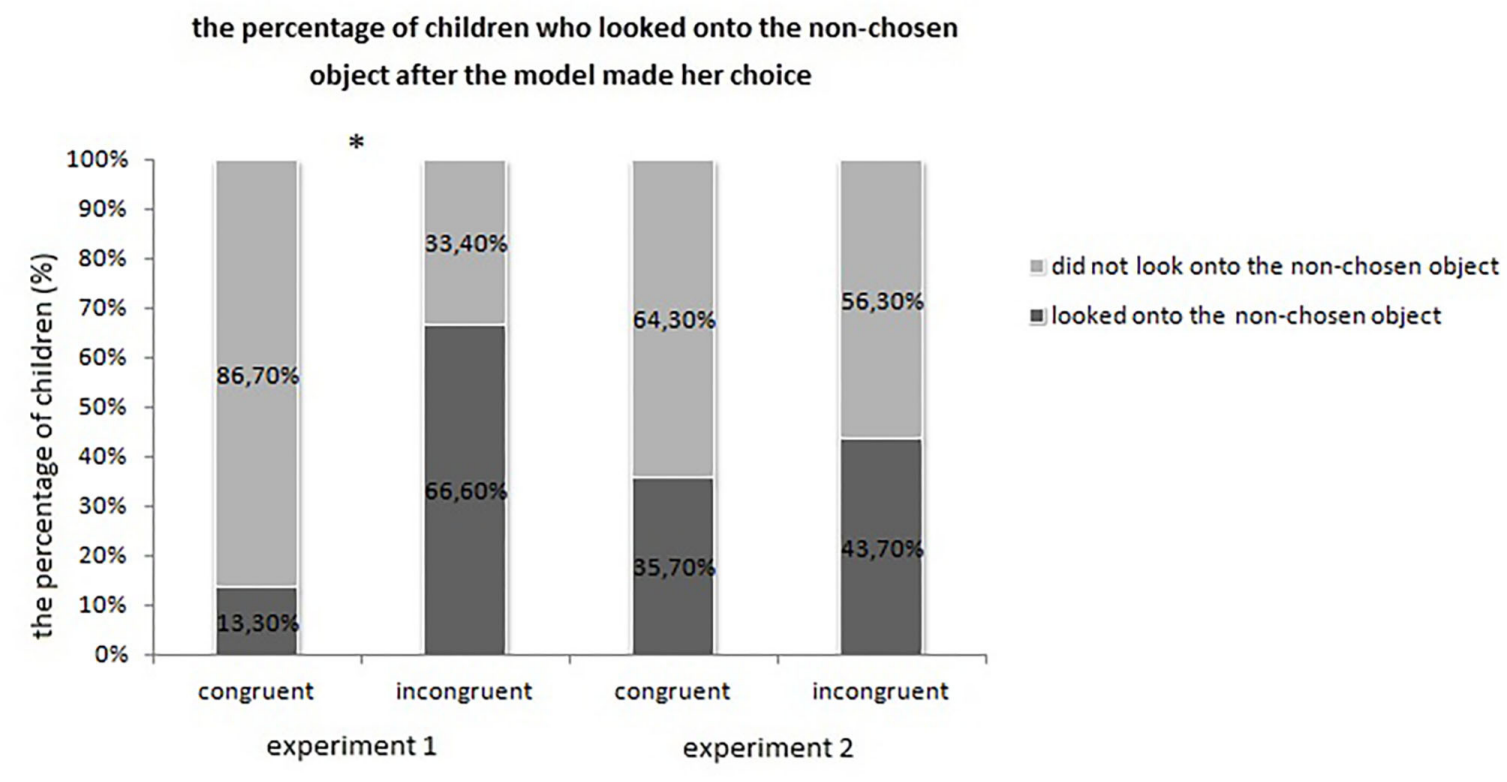
Looking patterns of experiment 1 and 2. This graph depicts the percentage of children who looked onto the non-chosen object after the model made her choice. The asterisk indicates the significant different which was detected in case of Experiment 1.

Participants taking part in the congruent condition watched the scene on average for 6.54 s (*SD* = 3.85 s) after the model had made her choice. In the incongruent condition, children did so for 9.54 s (*SD* = 1.08 s) on average. Mann Whitney U Test revealed that this difference was significant (*U* = 54; *p* = 0.015; Cohen's *d* = 1.061), which means that condition had a main effect on children's looking pattern after the choice was made by the model (see Figure 3). Further analyses revealed that 2 children out of 15 looked over the non-chosen object, while 10 children out of the 15 did so in the incongruent condition (see Figure 4). Based on a Pearson Chi-Square Test, the difference was significant [χ^2^(1) = 8,889, *p* = 0.003; *OR* = 0.076].

To confirm our results we also performed analyses with the JASP statistical software program. Bayesian independent samples *T*-test confirmed the substantial difference in children's looking times between the conditions after the model made her choice (*BF10* = 6.555). In addition, it revealed a reliable difference in the number of the children between the two conditions who looked over to the non-chosen object (*BF10* = 18.652).

#### Interim Conclusion

The results of Experiment 1 indicated that children were surprised about the model's choice in the incongruent condition, while they were not in the congruent one. They looked at the video for a longer period of time and they also tended to look over to the non-chosen object more frequently, which suggest that they formed expectations which were not entirely fulfilled. In light of these results it seems that children at the age of two expect native speakers to use the same tool for the same goal.

For creating specific expectations of others' behavior, children should possess sufficient information of the “others.” For example, they may expect native speakers to make enduring tool-function mappings because they know that objects usually have one certain function in their culture. On the other hand, they may suspend these expectations for someone who belongs to another culture in the absence of any relevant information of the culture specific norms, habits and behaviors of that group. Although the teleo-functional stance is a basic element of human cognition, it develops during the first years of life (Casler and Kelemen, 2007) which means learning—and especially cultural learning—could take an important role in the process. Consequently it is feasible that children may not necessarily expect out-group members to similarly form a consensus about the normative usage of objects. In that case children may expect out-group members to use an object based merely on its affordance. On the other hand, it is also conceivable, that children also expect out-group members to act alike, even if they do not possess any specific knowledge regarding the norms or habits of that specific group. To shed light on what assumption children have for out-group members' behavior we created Experiment 2, in which we used the very same procedure as we used in Experiment 1 but in the test phase the model spoke in a foreign language.

Furthermore, Experiment 2 will also help to disambiguate the question whether the results of Experiment 1 stem from a pure in-group preference *per se* or not. It is feasible that children looked at the not chosen object in the incongruent condition due to a mere in-group preference, since that object was previously used by the native speaker person. With the help of Experiment 2 we can explore this possibility in more detail. If in-group preference led children to look more frequently onto the non-chosen object in Experiment 1, the same looking pattern should appear irrespectively of the model's spoken language in the test phase in the following experiment as well.

## Experiment 2

### Materials and Method

#### Participants

Thirty monolingual Hungarian caucasian children (14 girls and 16 boys) between the age of 20 and 28 months (mean age = 23,5 months; *SD* = 0,97 months) took part in the study of whom 14 children (mean age = 23,3 months; *SD* = 0,27 months) took part in the congruent condition while 16 (mean age= 23,68 months; *SD* = 0,26 months) in the incongruent condition. An additional two children were tested but excluded due to calibration error. The parents of the participants signed informed consent before the experiment.

#### Equipment, Stimuli and Procedure

The equipment, stimuli and procedure were identical to the ones used in Experiment 1. The only difference was that during the test phase the person said the above mentioned sentence (“My name is Olga, I am 25 years old.”) in a foreign (Russian) language. This difference did not have an effect on the video stimuli's length or appearance.

## Results and Discussion

The procedure of the data analysis was the same as mentioned above in Experiment 1. On average children watched the familiarization phase for 34.9 s (*SD* = 5.44 s) on which condition did not have an effect (*U* = 100; *p* = 0.618, Cohen's *d* = 0.141). During the test phase, after the model had chosen, toddlers on average watched the stimuli for 7.47 s (*SD* = 3.41 s) in the congruent condition, while they did so for 8.5 s (*SD* = 2.84 s) in the incongruent condition, which did not differ significantly from each other (*U* = 68.5; *p* = 0.07; Cohen's *d* = 0.328) (see Figure 3). Further analyses revealed that 5 out of 14 children looked at the non-chosen object in the congruent condition and 7 out of 16 children did so in the incongruent condition, which difference was not significant either based on a Pearson Chi-Square test [χ^2^(1) = 0,201, *p* = 0.654; *OR* = 0.7143] (see Figure 4).

The same results were revealed with a Bayesian independent samples *t*-test. Children looked at the stimuli equally long after the model had made her choice (*BF10* = 0.467) and equal percentage of children looked onto the not chosen object in both conditions (*BF10* = 0.371). According to these results, children's looking pattern was not affected by whether the object had previously been used by the foreign or the native speaker model. In Experiment 2, children did not look longer in either conditions and they did not look over to the not chosen object more frequently when it had previously been used by the native speaker person. Children did not show any, either in-group or out-group, preference in Experiment 2.

To sum up, the results of Experiment 2 indicate that children did not form expectations about foreign speakers' behaviors regarding tool usage, or they expected them to choose between the objects based on the objects' affordance which was equivalent in the present study.

### General Discussion

According to recent studies language has a dominant role in social categorization. The significance of language as a marker of category boundaries suggests that children from a young age attend to more than simple perceptual cues of similarity when sorting the social world and lend significance to behavioral indicators as well (or even primarily). Acting alike and conformity of behavior is an important element of group living and studies suggest that such expectations about the behavior of group members arise early in development (Powell and Spelke, 2013). Moreover, children seem to be motivated to align their own behavior with those of linguistic in-group members as shown by the findings of selective imitation studies (e.g., Buttelmann et al., 2013). Evidence suggest that this epistemic function of language based social categorization is underlain by early emerging assumptions that group members not only act alike but possess shared knowledge in various domains as well (Oláh et al., 2014; Soley and Spelke, 2016; Soley and Aldan, 2020).

With the help of our first experiment, we demonstrated that already two-year-old children do have expectations of native speakers' commonly shared cultural practices, like tool usage. After observing two function demonstrations of two unfamiliar tools, which were identically appropriate for slicing an apple, children expected native speakers to use the same object for the same goal. This result suggests that children expect native speakers to act in the same manner. We assume that these expectations stem not only from the pure expectations of “similar people should act in the same way,” but from children's propensity to assume that similar behavioral patterns are motivated by similar knowledge states that are represented as commonly shared cultural knowledge. This interpretation of the results was supported with the results of Experiment 2 as well. In our second experiment, we repeated the very same procedure as in Experiment 1 with the exception that during the test phase the person spoke in a foreign language. We were interested in whether children would form similar expectations about out-group members as they did about in-group members. In Experiment 2, in contrast to Experiment 1, there was no significant difference either between the looking times in the congruent and incongruent conditions, nor in the looking pattern at the chosen and non-chosen objects. Altogether these results indicated that children had little—if any—expectations in the case of the foreign speaker model, and they did not expect foreign speakers to act alike. Although it has been suggested that children do form expectations about non-native speakers too, based on our results this is not inevitably true. Liberman et al. (2016) demonstrated that infants before their first birthday expect foreign speakers to share food preferences (Liberman et al., 2016) and also expect foreign speakers to affiliate with each other rather than with someone who speaks another language (Liberman et al., 2016).

Presumably, culturally shared knowledge contains many opaque and arbitrary elements that are not necessarily known by someone who do not belong to the same group. Without sufficient information children should not have refined—if any—expectation of the content of unfamiliar cultural knowledge. The strategy then, to form clear expectations only for native speakers in the domain of tools may be adaptive, since two different cultures may treat tools in different ways. In light of these, children may form expectations about foreign speakers in relation to socially relevant events (e.g., affiliation, Liberman et al., 2016) that do not inevitably require culture dependent shared knowledge, but they do not form expectations about them in the domain of culture specific knowledge (e.g., functions of tools, Oláh et al., 2016; Peto et al., 2018). The expectation that people who speak the same language should affiliate with each other rather than with people who speak differently can be based on mere familiarity or similarity, but it does not require any culture specific knowledge content.

Nevertheless, while the mentioned studies by Liberman and the present experiment have similarities, they also differ in an important way: the applied methodology. In the studies of Liberman attitude was the aspect of behavior that differed or remained the same across protagonists, while in the present study results are based purely on tool usage. In our experiment the model prefers to use a tool without any facial expression, which leads to a solely functionally determined tool choice rather than a personal preference. In this sense the results of these studies are not necessarily comparable to each other, since children may interpret apparent emotional based behavior and pure norm-based behavior differently. Further studies are required to assay in depth on this discrepancy regarding the expectations toward foreign people's behaviors. It is feasible that children do have expectations of out-group members in some domain but they do not form expectations in other domains. In the future it would be interesting to shed light on this possibility in more detail.

Taken together, we suggest that the results of Experiment 1 and 2 indicate that children treat spoken language as an indicator of culturally shared knowledge and they expect native speakers not just to act alike, but also to share other aspects of the culture specific knowledge, for example the functions of tools. This interpretation of the data would also imply that two-year-old children expect native speakers to follow the so-called teleo-functional principles and treat function as an enduring and intrinsic property of the tool that inevitably determines a tool's usage. If children did not expect teleo-functional principles to be known by culturally in-group members, they could think people may use different tools for the same goals and should not be surprised when the native speakers use different tools for slicing an apple.

Note that a possible alternative explanation of the results would be that children prefer to attend to native speakers, or show stronger associations for them. These assumptions, however, would clearly suggest that children in the test phase should preferably attend the object which was previously used by the native speaker model, irrespective of the partner present, so the same pattern should have emerged in both of our experiments. While this pattern is there in Experiment 1, in Experiment 2 children did not look over to the not chosen object more frequently when it had previously been used by the native speaker model. Since we used the very same methodology in both experiments, we believe that the overall pattern of results cannot be explained by “ingroup preference” or mere association *per se*.

Furthermore, it may be argued that children's expectations are not pertinent to the knowledge states of the protagonists *per se* but arise from a simpler assumption that group members should behave in a similar manner, without appealing to underlying knowledge states. Since we did not directly test attributions of knowledge states, rather expectations about overt behavior, we cannot unambiguously differentiate between these two alternatives. However, we find it plausible to assume that these expectations about behavior are rooted in assumptions about knowledge states for several reasons. First, ample evidence suggests that children from an early age rely on mental state attributions when forming expectations about the behavior of others (e.g., Onishi and Baillargeon, 2005; Kovács et al., 2010). Second, if children merely relied on an expectation that group members should act alike, they should not suspend this expectation for out-group members (see Experiment 2). The study of Powell and Spelke (2013) also suggests that these assumptions are held for groups of which the child is not a member. Third, previous studies with different methodologies provide direct evidence that young children use linguistic group membership to make inferences about different aspects of shared knowledge as well (Oláh et al., 2014; Soley and Spelke, 2016; Peto et al., 2018).

The results of the present study are based on a violation of expectation paradigm and introduced gaze-alternation as a measure. While many studies utilized and approved the former looking time method, the gaze-alteration measure is novel. The results of these two methodology together—since applied as independent variables—suggests that gaze-alternation might be a reliable and interesting indicator of information gathering in eye-tracking studies. Obviously, this assumption should be investigated in the future.

Further studies should investigate the question whether children have assumptions of other pieces of culture specific knowledge (e.g.: arbitrary rules, normative behaviors) to be commonly shared by native speakers or not. Nevertheless, besides culture specific knowledge, age- and gender-related knowledge also forms essential parts of our everyday life. Consequently, they are also reliable sources of commonly accepted and followed rules and norms, and in their own domains, they can evoke epistemic trust. Later studies should explore the question whether children form epistemic assumptions of other social categories as well or not and whether acting alike (‘group members behave in the same manner) or knowing the same things (group members possess shared knowledge) are rooted in the same expectations or not, and what may be the differences between these two approaches.

The findings presented above also shed light on the possibility that the teleo-functional stance may not be a generally expected principle but instead a culture specific one. Eventhough, most of the societies apply the one tool for one purpose principle it seems that two-year-olds are not necessarily aware of its universality. Children did not use this principle in Experiment 2 for foreign speakers. They did not expect foreign speakers to use one specific tool for one specific purpose. In lack of sufficient information children may rely mainly on the perceived affordance properties, and do not apply the expectation that there is one function that is socially determined, or it is also possible, that they may think that in other cultures, tools may be used for multiple purposes create a tool for being used for multiple functions. In the future, it would be interesting to explore this question in detail. When and why do we start to treat the teleo-functional principle as a universally valid axiom, (if ever)? Objects with multiple functions would be more efficient, especially in poor cultures (e.g., in developing countries) where there is less possibility to make and possess hundreds of objects. Would we expect those cultures to create one specific tool for one specific function or in awareness of their circumstances would we override the teleo-functional principle and treat it as culture specific rather than generally human specific?

## Data Availability Statement

The raw data supporting the conclusions of this article can be found online at: https://osf.io/natdb/?view_only=2c1b0b1189e348bc86a3ab06e6e75285.

## Ethics Statement

The studies involving human participants were reviewed and approved by Ethical Research Committee of University Eötvös Loránd, Budapest, Hungary. Written informed consent to participate in this study was provided by the participants' legal guardian/next of kin.

## Author Contributions

All authors listed have made a substantial, direct and intellectual contribution to the work, and approved it for publication.

## Conflict of Interest

The authors declare that the research was conducted in the absence of any commercial or financial relationships that could be construed as a potential conflict of interest.

## Publisher's Note

All claims expressed in this article are solely those of the authors and do not necessarily represent those of their affiliated organizations, or those of the publisher, the editors and the reviewers. Any product that may be evaluated in this article, or claim that may be made by its manufacturer, is not guaranteed or endorsed by the publisher.
